# Supplementary material for: A biofilm-tropic Pseudomonas aeruginosa bacteriophage uses the exopolysaccharide Psl as receptor
Source: eLife. 2025 Aug 11;13:RP102352. doi: 10.7554/eLife.102352 (PMC12339003; doi:10.7554/eLife.102352)
Supplement: Supplementary file 1. — Strains and Plasmids. [file elife-102352-supp1.docx]

| **Table S1. Strains and Plasmids** | | |
| --- | --- | --- |
| Strain | genotype | Reference |
| RP1860  RP44 | PAO1F wild type  PA14 wild type | (1)  (2) |
| RP4708 | PAO1F *∆fliF* | this study |
| RP4126 | PAO1F *∆pilA* | this study |
| RP9170 | PAO1F *∆fliF ∆pilA* | this study |
| RP12980 | PAO1F *∆fliF2* | this study |
| RP13077 | PAO1F *∆pslC* | this study |
| RP13080 | PAO1F *∆fliF2 ∆pslC* | this study |
| RP13081 | PAO1F *∆fliF2 ∆pslD* | this study |
| RP13089 | PAO1F *∆wspF* | this study |
| RP3302 | PAO1F *∆fliC* | (3) |
| RP12922 | PAO1F *∆fliE* | this study |
| RP12894 | PAO1F *∆fliG* | this study |
| RP12935 | PAO1F *∆flgBCDEFG* | this study |
| RP12859 | PAO1F *∆flgI* | this study |
| RP12936 | PAO1F *flhA(R147A)* | this study |
| RP12897 | PAO1F *∆cheA* | this study |
| RP12896 | PAO1F *∆fliHIJ* | this study |
| RP12923 | PAO1F *∆flhA* | this study |
| RP12911 | PAO1F *∆fliOPQRflhB* | this study |
| RP1865 | PAO1F *∆fleQ* | (4) |
| RP13249 | PAO1F *∆fliF2 ∆fleQ* | this study |
| RP12961 | PAO1F ∆*fliF* ∆*gmd* | this study |
| RP12963 | PAO1F ∆*fliF* ∆*wbpM* | this study |
| RP13195 | PAO1F ∆*fliF2* ∆*ssg* | this study |
| DH5α | F– *endA1 glnV44 thi-1 recA1 relA1 gyrA96 deoR nupG purB20 φ80dlacZΔM15 Δ(lacZYA-argF)U169, hsdR17(rK–mK+), λ–* |  |
| SM10 λ*pir* | *thi thr leu tonA lacY supE recA*::RP-2Tc::Mu *kanR*::λ*pir* | (5) |
|  |  |  |
| plasmid | genotype | Reference |
| pPSV37 | *colE1* origin, *gentR*, PA origin, *oriT, p_lac_*_UV5_ promoter, *lacIq* | (6) |
| pP37-*fliF* | *fliF* under control of *p_lac_*_UV5_ promoter | this study |
| pJN105 | *gentR*, *p_BAD_* promoter, *araC, pBBR1* origin | (7) |
| pJN2133 | pJN105 with PA2133 under control of *p_BAD_* | (8) |
| pP25-GFPo | constitutively expressed GFPmut3, *bla* | (9) |
| pPSV35-CV | *colE1* origin, *gentR*, PA origin, *oriT, p_lac_*_UV5_, *lacIq,VSV-G tag* | (10) |
| pPSV35-CV-*pslC* | *pslC-VSV-G* under control of *p_lac_*_UV5_ promoter | Joseph Mougous, unpublished |
| pPSV35-CV-*pslD* | *pslD-VSV-G* under control of *p_lac_*_UV5_ promoter | Joseph Mougous, unpublished |
| pEXG2 | allelic exchange vector, *colE1* origin, *oriT*, *gentR*, *sacB* | (11) |
| pEXG2-*∆pilA* | *pilA* deletion removing codons 3-148, in pEXG2 | this study |
| pEXG2-*∆fliF* | *fliF* deletion removing codons 3-597, in pEXG2 | this study |
| pEXG2-*∆fliF2* | *fliF* deletion removing codons 65-490, in pEXG2 | this study |
| pEXG2-*∆pslC* | *pslC* deletion removing codons 8-297, in pEXG2 | this study |
| pEXG2-*∆pslD* | *pslD* deletion removing codons 14-247, in pEXG2 | this study |
| pEXG2-*∆fliE* | *fliE* deletion removing codons 3-108, in pEXG2 | this study |
| pEXG2-*∆fliG* | *fliG* deletion removing codons 1-339, in pEXG2 | this study |
| pEXG2-*∆fliHIJ* | *fliHIJ,* clean deletion moving *fliJ* STOP to *fliG*, in pEXG2 | this study |
| pEXG2-*∆fliOPQRflhB* | deletes *fliO*(codon 2)-*flhB*(codon 377), in pEXG2 | this study |
| pEXG2-*∆flgBCDEFG* | deletes *flgB*(codon 1)-*flgG*(codon 253), in pEXG2 | this study |
| pEXG2-*∆flgI* | *flgI* deletion removing codons 3-368, in pEXG2 | this study |
| pEXG2-*∆flhA* | *flhA* deletion removing codons 4-704, in pEXG2 | this study |
| pEXG2-*flhA(R147A)* | *flhA* R147A mutation, in pEXG2 | this study |
| pEXG2*-∆fleQ* | *fleQ* deletion removing codons 4–487, in pEXG2 | (4) |
| pEXG2-*∆wspF* | *wspF* deletion removing codons 11-329, in pEXG2 | this study |
| pEXG2-*∆cheA* | *cheA* deletion removing codons 3-752, in pEXG2 | this study |
| pEXG2-∆*gmd* | *∆gmd*, clean deletion moving *rmd* stop to *gmd* stop | this study |
| pEXG2-∆*wbpM* | *wbpM* deletion removing codons 3-646, in pEXG2 | this study |
| pEXG2-∆*ssg* | *ssg* deletion, in pEXG2 | this study |

1. Bleves S, Soscia C, Nogueira-Orlandi P, Lazdunski A, Filloux A. Quorum sensing negatively controls type III secretion regulon expression in Pseudomonas aeruginosa PAO1. J Bacteriol. 2005;187(11):3898-902.

2. Rahme LG, Stevens EJ, Wolfort SF, Shao J, Tompkins RG, Ausubel FM. Common virulence factors for bacterial pathogenicity in plants and animals. Science. 1995;268(5219):1899-902.

3. Sun Y, Karmakar M, Roy S, Ramadan RT, Williams SR, Howell S, et al. TLR4 and TLR5 on corneal macrophages regulate Pseudomonas aeruginosa keratitis by signaling through MyD88-dependent and -independent pathways. J Immunol. 2010;185(7):4272-83.

4. Tomalka AG, Stopford CM, Lee PC, Rietsch A. A translocator-specific export signal establishes the translocator-effector secretion hierarchy that is important for type III secretion system function. Mol Microbiol. 2012;86(6):1464-81.

5. Miller VL, Mekalanos JJ. A novel suicide vector and its use in construction of insertion mutations: osmoregulation of outer membrane proteins and virulence determinants in Vibrio cholerae requires toxR. J Bacteriol. 1988;170(6):2575-83.

6. Lee PC, Stopford CM, Svenson AG, Rietsch A. Control of effector export by the Pseudomonas aeruginosa type III secretion proteins PcrG and PcrV. Mol Microbiol. 2010;75(4):924-41.

7. Newman JR, Fuqua C. Broad-host-range expression vectors that carry the L-arabinose-inducible Escherichia coli araBAD promoter and the araC regulator. Gene. 1999;227(2):197-203.

8. Hickman JW, Tifrea DF, Harwood CS. A chemosensory system that regulates biofilm formation through modulation of cyclic diguanylate levels. Proc Natl Acad Sci U S A. 2005;102(40):14422-7.

9. Goodman AL, Kulasekara B, Rietsch A, Boyd D, Smith RS, Lory S. A signaling network reciprocally regulates genes associated with acute infection and chronic persistence in Pseudomonas aeruginosa. Dev Cell. 2004;7(5):745-54.

10. Hood RD, Singh P, Hsu F, Guvener T, Carl MA, Trinidad RR, et al. A type VI secretion system of Pseudomonas aeruginosa targets a toxin to bacteria. Cell host & microbe. 2010;7(1):25-37.

11. Rietsch A, Vallet-Gely I, Dove SL, Mekalanos JJ. ExsE, a secreted regulator of type III secretion genes in Pseudomonas aeruginosa. Proc Natl Acad Sci U S A. 2005;102(22):8006-11.
