# Supplementary material for: A biofilm-tropic Pseudomonas aeruginosa bacteriophage uses the exopolysaccharide Psl as receptor
Source: eLife. 2025 Aug 11;13:RP102352. doi: 10.7554/eLife.102352 (PMC12339003; doi:10.7554/eLife.102352)
Supplement: Supplementary file 2. — Primers. [file elife-102352-supp2.docx]

| **Table S2. Primers** | | |
| --- | --- | --- |
| Name | Sequence (5’->3’) | use |
| fliF-5-1 | AAAAAGAATTCACGGACCCTGCAGCGGTCGCGGGCAT | *∆fliF*, EcoRI |
| fliF-5-2 | AACTCGAGCCGCAAGCATGCTGAAGGCCATGAACTAGTTATCCTCGCGCCGCT |  |
| fliF-3-1 | TTCAGCATGCTTGCGGCTCGAGTTGAGTAAGCGCCATGAGTGAGAATCGT |  |
| fliF-3-2 | AAAAAAAGCTTGTTCAGCGAGGAGACGCGCAGGACGAT | *∆fliF*, HindIII |
| fliF2-5-2 | CACGCCGAGCACCTGCTTGACGATCTGCTGGGACCAGAGCACGACG | *∆fliF2* |
| fliF2-3-1 | GTCGTGCTCTGGTCCCAGCAGATCGTCAAGCAGGTGCTCGGCGTG |  |
| FliF-5G | TGACCATGATTACGAATTGTAGCTAGCTAGGAATTCCGGCGCGAGGATAACTAGTTCATG | Gibson clone *fliF* |
| FliF-3G | CCCGTTTAGAGGCCCCAAGGGGTTATGCTAAAGCTTTTACTCATCGGCGTTGATCCACTC | into pPSV37 EcoRI/HindIII |
| pilA-5-1 | TTCAGCATGCTTGCGGCTCGAGTTTTTCATGAATCTCTCCGTTGATTAT |  |
| pilA-5-2 | AAAAAGAATTCGCGGGGTCGGAGATGCCTACAAA | *∆pilA*, EcoRI |
| pilA-3-1 | AAAAATCTAGACGGTATCGACCGGGCAATTGCCGA | *∆pilA*, XbaI |
| pilA-3-2 | AACTCGAGCCGCAAGCATGCTGAAAACTAAGGTGATCGAAGGTGGCTT |  |
| pslC-5-1 | GCACGATCATGCGCACCCGTGGAAATTAATTAAGGTACCCGCGCAGCCAGGACGTCAAG | *∆pslC*, |
| pslC-5-2 | CTTCCAGTAGCCTGGAAACAGGGAATGACCAGGGCGCAGC | Gibson clone |
| pslC-3-1 | GCTGCGCCCTGGTCATTCCCTGTTTCCAGGCTACTGGAAG | into pEXG2 |
| pslC-3-2 | TTATACGAGCCGGAAGCATAAATGTAAAGCAAGCTTGGATCGCCTGCTCCATGTTG | KpnI/HindIII |
| pslD-5-1 | ACGATCATGCGCACCCGTGGAAATTAATTAAGGTACCCCTCGGAACTGGGGATCAAGACC | *∆pslD*, |
| pslD-5-2 | CATTGTTGACGGTGTAGCTGACCAGGGCGAGCATGGCGAGC | Gibson clone |
| pslD-3-1 | CTCGCCATGCTCGCCCTGGTCAGCTACACCGTCAACAATG | into pEXG2 |
| pslD-3-2 | TTATACGAGCCGGAAGCATAAATGTAAAGCAAGCTTGGTGAAGCTGATCTCCATCAC | KpnI/HindIII |
| fliE-5-1 | CGATCATGCGCACCCGTGGAAATTAATTAAGGTACCCACCCCGCCGAGCGTCGAGATTCC | *∆fliE*, |
| fliE-5-2 | CTAGTTATCCTCGCGCCGCTCAGACACTCATGACTCTTCCTCCAACAGCC | Gibson clone |
| fliE-3-1 | GGCTGTTGGAGGAAGAGTCATGAGTGTCTGAGCGGCGCGAGGATAACTAG | into pEXG2 |
| fliE-3-2 | ATTATACGAGCCGGAAGCATAAATGTAAAGCAAGCTTCGAAACCGACGTTGTTGTCGGTC | KpnI/HindIII |
| fliG-5-1 | GATCATGCGCACCCGTGGAAATTAATTAAGGTACCGAAGACCGGCGAGGTCAGCCACCAG | *∆fliG*, |
| fliG-5-2 | CTTTGTCCTTGTCGTGGGGGACCATTACTCATCGGCGTTGATCCAC | Gibson clone |
| fliG-3-1 | GTGGATCAACGCCGATGAGTAATGGTCCCCCACGACAAGGACAAAG | into pEXG2 |
| fliG-3-2 | TTATACGAGCCGGAAGCATAAATGTAAAGCAAGCTTCGCCCATCGGCAGCAGCTTCAGAG | KpnI/HindIII |
| fliH-5-1 | GATCATGCGCACCCGTGGAAATTAATTAAGGTACCGTCTCCTCGCTGAACACCGTGCAG | *∆fliHIJ*, |
| fliH-5-2 | GTCGCGTCCGTTCTTCTGAGTCGTCAGATCATCTCCTCGCCACCCTTG |  |
| fliJ-3-1 | CAAGGGTGGCGAGGAGATGATCTGACGACTCAGAAGAACGGACGCGAC |  |
| fliJ-3-2 | ATACGAGCCGGAAGCATAAATGTAAAGCAAGCTTGTGGCTGGTACATCGGCAACTGGACC |  |
| fliO-5-1 | CGATCATGCGCACCCGTGGAAATTAATTAAGGTACCTGGCGCTACAGATTCTCGAAGC | *∆fliOPQRflhB*, |
| fliO-5-2 | GCGTAGCTCCAGCCAGGTCACTCCGCATGTCAGCGCAGCTTCTTGATG | Gibson clone |
| flhB-3-1 | CATCAAGAAGCTGCGCTGACATGCGGAGTGACCTGGCTGGAGCTACGC | into pEXG2 |
| flhB-3-2 | ATTATACGAGCCGGAAGCATAAATGTAAAGCAAGCTTGATCCGTCCGGCGACCACATCGG | KpnI/HindIII |
| flgB-5-1 | AGCACGATCATGCGCACCCGTGGAAATTAATTAAGGTACCTCCGGCGCCTATGTGCACTTCGACATG | *∆flgBCDEFG,* |
| flgB-5-2 | CAAAGATTCTGGGTGACGAAGGACAAGGCTGCGAACCTGTTTGCCGGTGTG | Gibson clone |
| flgG-3-1 | CACACCGGCAAACAGGTTCGCAGCCTTGTCCTTCGTCACCCAGAATCTTTG | into pEXG2 |
| flgG-3-2 | CACATTATACGAGCCGGAAGCATAAATGTAAAGCAAGCTTGATCGAACCGGTCAGGCTGTTGCTCTGC | KpnI/HindIII |
| flgI-5-1 | AGCACGATCATGCGCACCCGTGGAAATTAATTAAGGTACCCTTCCGCGTGGGCGACATCATCAC | *∆flgI*, |
| flgI-5-2 | GAATCCATGGCGTCGTCCTCAAATGGTCATCGCGAGCGTCCTCAGAAC | Gibson clone |
| flgI-3-1 | GTTCTGAGGACGCTCGCGATGACCATTTGAGGACGACGCCATGGATTC | into pEXG2 |
| flgI-3-2 | CACATTATACGAGCCGGAAGCATAAATGTAAAGCAAGCTTAGGCGCCTCTGGTTGAGCAGCTTC | KpnI/HindIII |
| flhA-5-1 | CGATCATGCGCACCCGTGGAAATTAATTAAGGTACCCCGCTTTCGCTGGGACTGAGCCTG | *∆flhA*, |
| flhA-5-2 | CGCAGCCCTCGTTCAGTTCTGTCCGCGATCCACTCTCGACTCCCCTGC | Gibson clone |
| flhA-3-1 | GCAGGGGAGTCGAGAGTGGATCGCGGACAGAACTGAACGAGGGCTGCG | into pEXG2 |
| flhA-3-2 | TATACGAGCCGGAAGCATAAATGTAAAGCAAGCTTGGCCCCAGGCGATGGAACCCAGTTG | KpnI/HindIII |
| flhAR147A-5-1 | GATCATGCGCACCCGTGGAAATTAATTAAGGTACCCGCGCATTCCGGCGTCAAAAG | *flhA(R147A)*, |
| flhAR147A-5-2 | GAAGCGCGCGCTGACTTCGGAAATGGCCCCGGCGCCCTTGGTCACCACCAC | Gibson clone |
| flhAR147A-3-1 | GTGGTGACCAAGGGCGCCGGGgccATTTCCGAAGTCAGCGCGCGCTTCAC | into pEXG2 |
| flhAR147A-3-2 | TTATACGAGCCGGAAGCATAAATGTAAAGCAAGCTTGCCGAGGCCGATGAAGGAAACATG | KpnI/HindIII |
| flhAR147A-Test | CGCGCTGACTTCGGAAATggc | + with 5-1 primer and mutation |
| wspF-5-1 | GCACGATCATGCGCACCCGTGGAAATTAATTAAGGTACCCCGGCGCCTTGCTGGACGACG | *∆wspF*, |
| wspF-5-2 | CTAATCGAATACCTCCGCCAGCGGCATGTCATTGACGATTCC | Gibson clone |
| wspF-3-1 | GGAATCGTCAATGACATGCCGCTGGCGGAGGTATTCGATTAG | into pEXG2 |
| wspF-3-2 | TTATACGAGCCGGAAGCATAAATGTAAAGCAAGCTTGATGGCGTCCGGCAGCTTGACC | KpnI/HindIII |
| cheA-5-1 | GATCATGCGCACCCGTGGAAATTAATTAAGGTACCCAGTTGTCCTCGCAGCTCAATGAC | *∆cheA*, |
| cheA-5-2 | CGGAAACCCATACGCGGCGTCAGATGCTCATTCGGCTGCTCCCAGAGACGTGTTAC | Gibson clone |
| cheA-3-1 | GTAACACGTCTCTGGGAGCAGCCGAATGAGCATCTGACGCCGCGTATGGGTTTCCG | into pEXG2 |
| cheA-3-2 | ATACGAGCCGGAAGCATAAATGTAAAGCAAGCTTCATGGCTGGACGAGGTGGCCGAAG | KpnI/HindIII |
| gmd-3-1 | ATTATACGAGCCGGAAGCATAAATGTAAAGCAAGCTTCTGGTCGCCCAGCTCGATATAGC | ∆*gmd* |
| gmd-3-2 | GACTGGGAGTCACGGGTACGAGAAGAGTGAGCCATGCTGATTCCCGTGGTGCTTTCCGGC | Gibson clone |
| gmd-5-1 | GCCGGAAAGCACCACGGGAATCAGCATGGCTCACTCTTCTCGTACCCGTGACTCCCAGTC | into pEXG2 |
| gmd-5-2 | GACAGGAGCACGATCATGCGCACCCGTGGAAATTAATTAAGAGTCGCTGTGCCTGCAGTG | no A-band LPS (1) |
| wbpM-3-1 | ATTATACGAGCCGGAAGCATAAATGTAAAGCAAGCTTCTGCGCGCCCGGTTCTTCTCCAG | ∆*wbpM* |
| wbpM-3-2 | CTATTGAACGGGGCTGATAAATAGGATGTTGTATGCGCCTGACGGTGAAATCGTCGACTG | Gibson clone |
| wbpM-5-1 | CAGTCGACGATTTCACCGTCAGGCGCATACAACATCCTATTTATCAGCCCCGTTCAATAG | into pEXG2 |
| wbpM-5-2 | GACAGGAGCACGATCATGCGCACCCGTGGAAATTAATTAATAGGTATCGACGGTGCTGTG | No B-band LPS (2) |
| PA5001-5-1 | GCACGATCATGCGCACCCGTGGAAATTAATTAAGGTACCCATTTCGATCCGCACCCGGAC | ∆*ssg* |
| PA5001-5-2 | CGTCGCCAGGTCTTCTCCAGCTCTTTCTGGACCAGAAACAGAAC | Gibson clone |
| PA5001-3-1 | CTGTTTCTGGTCCAGAAAGAGCTGGAGAAGACCTGGCGACG | into pEXG2 |
| PA5001-3-2 | TTATACGAGCCGGAAGCATAAATGTAAAGCAAGCTTCACCTGTTTCTCCAGGCGTTGC | LPS outer core mutant (3) |
| Mar1x | GGGAATCATTTGAAGGTTGGTAC | 1^st^ rnd. TnSeq |
| olj376 | GTGACTGGAGTTCAGACGTGTGCTCTTCCGATCTGGGGGGGGGGGGGGGG | 1^st^ rnd. TnSeq |
| Mar2-InSeq | AATGATACGGCGACCACCGAGATCTACACCATTTAATACTAGCGACGCCATCTATGTGTCAG | 2^nd^ rnd. TnSeq |
| TdT_Index_1 | CAAGCAGAAGACGGCATACGAGATCGTGATGTGACTGGAGTTCAGACGTGTGCTCTTCCGATCT | 2^nd^ rnd. TnSeq |
| TdT_Index_2 | CAAGCAGAAGACGGCATACGAGATACATCGGTGACTGGAGTTCAGACGTGTGCTCTTCCGATCT |  |
| TdT_Index_3 | CAAGCAGAAGACGGCATACGAGATGCCTAAGTGACTGGAGTTCAGACGTGTGCTCTTCCGATCT |  |
| TdT_Index_4 | CAAGCAGAAGACGGCATACGAGATTGGTCAGTGACTGGAGTTCAGACGTGTGCTCTTCCGATCT |  |
| MarSeq2 | GTCAGACCGGGGACTTATCAGCCAAC | sequencing |

1. King JD, Poon KKH, Webb NA, Anderson EM, McNally DJ, Brisson JR, et al. The structural basis for catalytic function of GMD and RMD, two closely related enzymes from the GDP-D-rhamnose biosynthesis pathway. FEBS J. 2009;276(10):2686-700.

2. Creuzenet C, Lam JS. Topological and functional characterization of WbpM, an inner membrane UDP-GlcNAc C6 dehydratase essential for lipopolysaccharide biosynthesis in Pseudomonas aeruginosa. Mol Microbiol. 2001;41(6):1295-310.

3. Veeranagouda Y, Lee K, Cho AR, Cho K, Anderson EM, Lam JS. Ssg, a putative glycosyltransferase, functions in lipo- and exopolysaccharide biosynthesis and cell surface-related properties in Pseudomonas alkylphenolia. FEMS Microbiol Lett. 2011;315(1):38-45.
